# Intravesical Instillation of Kangfuxin Liquid Combined with Thrombin and Epidermal Growth Factor for Radiation-induced Hemorrhagic Cystitis in Patients with Cervical Cancer: A report of 34 cases

**DOI:** 10.1080/21655979.2021.1882141

**Published:** 2021-02-28

**Authors:** Qin Hu, Guihao Ke

**Affiliations:** Department of Gynecology, Fudan University Shanghai Cancer Center, Shanghai, China

**Keywords:** Radiation-induced hemorrhagic cystitis, intravesical instillation, Kangfuxin liquid, cervical cancer

## Abstract

This study aimed to assess the effectiveness and safety of intravesical instillation treatment of Kangfuxin liquid (KFL) combined with thrombin and epidermal growth factor (EGF) for radiation-induced hemorrhagic cystitis (HC) in patients with cervical cancer. A total of 34 patients with radiation-induced HC of grade 2–4 were treated with intravesical instillation of KFL combined with thrombin and EGF until the complete disappearance of hematuria and lower urinary tract symptoms (LUTS). Gentamicin was added if white blood cells were detected and bacterial culture was positive in the urine. All patients were followed up for 2 years to evaluate the clinical efficacy and safety of the treatment regimen. Patients with and without recurrent hematuria (n = 3, 9% and n = 31, 91%, respectively) were completely recovered from hematuria and LUTS by intravesical instillation treatment for 6–22 days. No adverse event was reported during the treatment and the 2-year follow-up for all patients. Thus, intravesical instillation of KFL combined with thrombin and EGF is an effective and safe therapeutic regimen for radiation-induced HC of grade 2–4 in patients with cervical cancer.

## Graphical abstract

**Figure:**
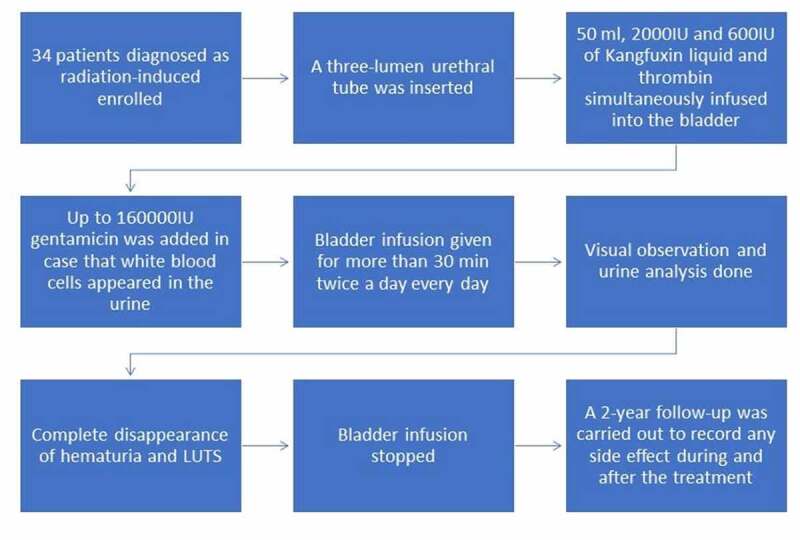

## Introduction

Cervical cancer is the most common gynecological cancer among females worldwide [1]. Up to 60% of the cervical cancer patients are indicated to receive pelvic radiotherapy [2]. Pelvic radiation can lead to short-or long-term damage to the bladder which is referred to as radiation cystitis (RC) or radiation-induced hemorrhagic cystitis (HC). Chronic HC occurs in up to 5% of the patients post pelvic radiotherapy [3] with microscopic or gross hematuria and lower urinary tract symptoms (LUTS) as the main clinical manifestations [4]. Intractable HC severely affects the quality of life of patient and sustained hematuria can even cause life-threatening hypovolemic shock [5,6].

The pathogenesis of RC is not fully understood which makes this recognized complication of pelvic radiotherapy difficult to treat. Common treatment options for radiation-induced HC include hyperbaric oxygen therapy (HBOT), clot evacuation, endoscopic fulguration, intravesical instillation with various agents, transurethral electrocoagulation, and surgery [7]. However, there are no standardized guidelines [8] and overtreatment or undertreatment often leads to a relapse of recurrence of hematuria and various side effects.

*Periplaneta americana* (PA) or American cockroach has been recorded as a kind of traditional Chinese medicine (TCM) in Shen Nong’s Herbal Classic for a long history [9]. Modern medical researches demonstrated that PA extracts and preparations have a variety of pharmacological activities including antibacterial, anti-tumor, anti-inflamation, promoting tissue repair, and enhancing immunity [10]. A number of TCM including PA extract and preparations have been widely used in various clinical applications [10]. Among them, Kangfuxin liquid (KFL), whose main ingredient is ethanol extract of PA, has been used for the treatment of burns, wounds, and ulcers for years [11]. Remarkable effect of KFL in the prevention and treatment of different mucosa injuries caused by radiation has been reported in clinical practice [12,13]. More recently, intravesical instillation of KFL was used to treat HC after hemopoietic stem cell transplantation [14].

In the present study, we aim to investigate the effect and safety of intravesical instillation of KFL combined with thrombin and epidermal growth factor (EGF) for radiation-induced HC in patients with cervical cancer.

## Patients and methods

### Patients

The current study was conducted in the Department of Gynecology, Shanghai Cancer Center of Fudan University from June 2015 to January 2018. Patients were enrolled if they were diagnosed as radiation-induced HC by the medical history of pelvic radiation. In the acute phase, patients feel urinary urgency and bladder pain while in the chronic stage, an irritative syndrome is coupled with hematuria. Exclusion criteria was hematuria caused by other diseases, coagulation dysfunction, severe urinary tract infection, hepatic, and renal dysfunction. As a result, a total of 34 patients with cervical cancer aged 30–69 were recruited and received intravesical instillation of KFL combined with thrombin and EGF.

Institutional ethical committee clearance was taken before conducting the study.

### Treatment and follow-up

All patients received intravesical instillation immediately after detection of gross or microscopic hematuria. They were inserted a three-lumen urethral tube, through which 50 ml, 2000IU, and 600IU of Kangfuxin liquid (Kunming Sano Pharmaceutical Co., Ltd.), EGF (Shenzhen Huashengyuan Gene Engineering Development Co., Ltd.) and thrombin (Shandong Taibang Biological Products Co., Ltd.), respectively, were simultaneously infused into the bladder. Up to 160000IU gentamicin (Zhejiang Ruixin Pharmaceutical Co., Ltd.) was added in case that white blood cells appeared in the urine. The bladder infusion was reserved for more than 30 min and given twice a day every day until the complete disappearance of hematuria and LUTS was measured by visual observation and then urinalysis. Hematuria was diagnosed by visually checking for the color and presence of blood clots in urine. Urine analysis included checking for WBC and bacteriologic culture.

### Evaluation of clinical efficacy and safety

According to our physical examination and patient symptoms in clinical practice of gynecology, the degree of radiation-induced HC was rated as grade 0–4 with a detailed description as the following: grade 0: no hematuria and no LUTS; grade 1: microscopic hematuria with or without LUTS; grade 2: gross hematuria with or without LUTS; grade 3: gross hematuria with a blood clot and LUTS and grade 4: urethral obstruction due to blood clot and severe LUTS.

Clinical efficacy of the treatment was described as the following: complete relief (CR): the disappearance of hematuria and LUTS (radiation-induced HC of grade 0); partial relief (PR): alleviated hematuria and LUTS; no effect (NR): hematuria and LUTS were not alleviated significantly.

A 2-year follow-up was carried out to record any side effect during and after the treatment. The safety evaluation was based on adverse events, which were defined and rated as described in Common Terminology Criteria for Adverse Events v5.0 (CTCAE v5.0) issued by the National Institutes of Health (NIH) and National Cancer Institute (NCI) on 27 November 2017.

## Results

This was a pioneer study done to investigate the effect and safety of intravesical instillation of KFL combined with thrombin and epidermal growth factor (EGF) for radiation-induced HC in patients with cervical cancer. This combination has not been previously used for treating this entity in the given disease setting.

### Patient characteristics

As shown in Table 1, a total of 34 patients with cervical cancer were eligible for our study, including radiation-induced HC of grade 2 (n = 3), grade 3 (n = 10) and grade 4 (n = 21). The mean age of patients was 49 ± 9.5 (30–69). All patients received radical radiation therapy, including external and intracavitary irradiation. The radiation dose for pelvic and local metastatic lymph node was 45–50.4 Gy and 58.8 Gy/28 f, respectively. The intracavitary brachytherapy was conducted with A point dose of 25 Gy–35 Gy/5-7 f. Summarily the mean radiation dose of patients was 80.4 ± 5.4 (75–88.8), corresponding to 88 ± 6.2 (82–97) of EDQ2 (Equivalent dose in 2 Gy fractions). Concurrent chemotherapy of cisplatin (40 mg/m^2^, qw for three to six times) or paclitaxel plus carboplatin (135 mg/m^2^ and AUC (area under the curve) 5q3w, respectively, for two to four times) were carried out for 28 (82%) and 6 (18%) patients, respectively. The gross hematuria observed in patients with cervical cancer was 20 ± 5.9 months (9–37) and the hemoglobin level before intravesical instillation treatment was 79.4 ± 16 g/L (43–110). A total of four patients received gentamicin, an aminoglycoside antibiotic, due to the presence of white blood cell (WBC) in the urine with positive bacteriological culture. All patients experienced LUTS such as pain, increased frequency, and urgency (data not shown).

**Table 1.** Baseline information of the patient with RC before treatment

Cancer type		Cervical cancer (n = 34)
Grade of HC	1	0
	2	3 (9%)
	3	10 (29%)
	4	21 (62%)
	Total	34
Age (years)	Mean±SD	49 ± 9.5
	Min-Max	30–69
Radiation dose (EDQ2, gy)	Mean±SD	88 ± 6.2
	Min-Max	82–97
Concurrent chemotherapy (n, %)	Cisplatin	28 (82%)
	Paclitaxel + Carboplatin	6 (18%)
Hemoglobin (g/L)	Mean±SD	79.4 ± 16.0
	Min-Max	43–110
Appearance of hematuria (months)	Mean±SD	20 ± 5.9
	Min-Max	9–37
WBC in urine with positive bacterical culture (n, %)		4 (12%)

### Clinical and safety evaluation

**Figure 1.**
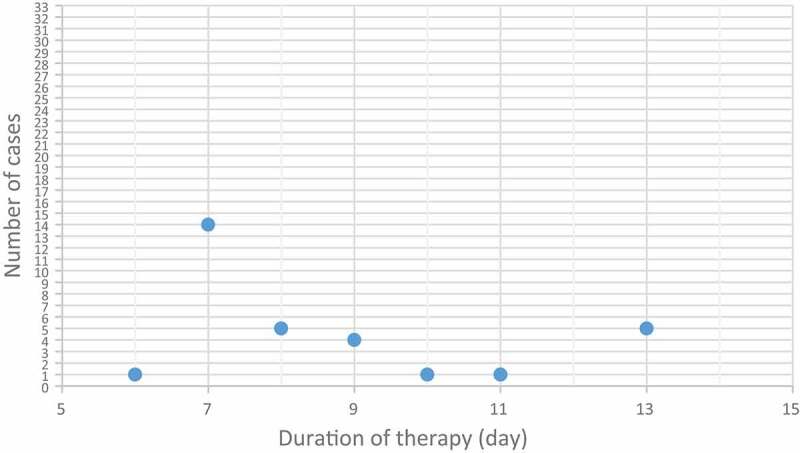
Number of patients with nonrecurrent gross and microscopic hematuria upon various time of intravesical instillation treatment

Hematuria and LUTS disappeared in 31 (91%) patients without recurrence after consecutive treatment for 6–13 days, as shown in Figure 1.

Recurrent hematuria occurred in three (9%) patients with cervical cancer. Gross hematuria was noticed by patient no. 19, 25, and 26 approximately 3, 6, and 6 months after the first treatment for 10, 12, and 13 days, respectively. These patients were given the secondary intravesical instillation of the same regimen for another 12, 7, and 7 days, respectively, until gross and microscopic hematuria disappeared.

Taken together, all the 34 patients with and without recurrent hematuria (3 and 31 patients, respectively) and LUTS achieved CR (100%) upon intravesical instillation treatment for 6–22 days.

No adverse event was reported during the treatment and 2-year follow-up.

## Discussion

Radiation-induced HC is one of the common complications after radiotherapy for pelvic tumors. Although various treatment options are available for patients with radiation-induced HC, there is no standardized guideline in clinical practice due to different patient conditions. Intravesical instillation is one of the most commonly used treatments for persistent hematuria because of its low cost, simplicity of operation, and availability in outpatient service. In the current study, radiation-induced HC of grade 2–4 in patients with cervical cancer received intravesical instillation treatment of KFL combined with thrombin and EGF.

It has been reported [15] that a glucosamine (GAG) layer located at the surface of the bladder wall plays a protective role against permeation by microorganisms, carcinogens, micro-crystals, and other drugs in the urine. High-energy radiation can damage the GAG layer and expose thin-walled blood vessels under the bladder mucosa directly to the urine, which can cause congestion, edema, hemorrhage, necrosis, and ulcer of bladder mucosaKFL used in the present study was made from the ethanol extract of PA, which contains compounds including cyclopeptide, diterpenoid, phenolic acid, fatty acids, and glycosides [16]. Most of the identified compounds have antibacterium, anti-inflammation, or enhancing immunity activities which can promote the wound-healing process [16]. In addition, thrombin functions as a hemostatic agent by converting fibrinogen into fibrin on wounds. It has also been proposed that the mechanism of wound healing from the KFL may be through the regulation of JAK/STAT3, PI3K/AKT, nuclear factor kappa B canonical pathway, and extracellular signal-regulated kinas signaling to affect cell proliferation, fibrogenesis, re-epithelialization, and remodeling. The compound periplanosides A-C can parallelly stimulate the production of human epidermal fibroblast collagen at a certain concentration. EGF can promote the migration of epithelial cells, granulocytes, and fibroblasts to the wound surface, thus shortening the time for wound healing. By intravesical instillation of KFL combined with thrombin and EGF, all the 34 patients with grade 2–4 radiation-induced HC were completely recovered. No adverse events related to the treatment were reported during a 2-year follow-up.

Generally, treatment strategy and total radiation dose depend on the location, size, and clinical stage of the tumor. The tolerance dose (TD5/5) of bladder tissue to radiation is 60 Gy. More than 10% of this radiation dose significantly increases the risk of radiation cystitis [17]. Although the advent of intensity-modulated radiation therapy may decrease radiation-induced bladder toxicity, robust data on long-term outcomes are limited [18]. In the present study, the radiation dose for cervical cancer patients varied from 82 to 97 Gy in order to achieve the goal of radical radiotherapy. All the 34 patients experienced gross hematuria and LUTS but achieved complete recovery by intravesical instillation of KFL combined with thrombin and EGF. The reason for recurrent hematuria (patient no. 19, 25, and 26, respectively) was not completely understood. However, although almost all the patients exhibit mild (90 g/L≤ hemoglobin < 100 g/L) ≥ to moderate (60 g/L≤ hemoglobin< 90 g/L) anemia due to radiation, the patient no. 19, 25, and 26 experienced severe anemia (30 g/L≤ hemoglobin< 60 g/L) with hemoglobin level at 56 g/L, 43 g/L, and 57 g/L, respectively. Our results suggest physicians may need to take hemoglobin level of patients into consideration when making a treatment schedule of intravesical instillation for radiation-induced HC.

Although the present study showed promising clinical efficacy and safety of intravesical instillation treatment using KFL combined with thrombin and EGF for grade 2 ~ 4 radiation-induced HC, other treatment options should also be carefully considered according to individual patient condition. Hyperbaric oxygen (HBO) is thought to be the only treatment to reverse the vascular radiation-induced pathophysiology [19] and used for patients with severe end-stage hemorrhagic radiation cystitis in addition to cystectomy. Yuan Shao et al. had discovered that intravesical instillation of hyaluronic acid (HA) was as effective in treating radiation-induced HC as HBO. It is well tolerated and resulted in a sustained decrease of bladder bleeding, pelvic pain and frequency of voiding for at least 12 months [20]. The characteristic of HA is to temporarily establish a GAG layer. For patients with severe bleeding, long-term regular perfusion is needed to obtain a long-term clinical efficacy and prevent a recurrence.

Taken together, we first reported the intravesical instillation treatment modality using KFL combined with thrombin and EGF for radiation-induced HC of grade 2–4 in patient with cervical cancer. A larger-sized prospective study and longer follow-up are needed to confirm the clinical efficacy and safety of this treatment regimen in the future.

## Conclusion

Intravesical instillation of KFL combined with thrombin and EGF is an effective and safe therapeutic regimen for radiation-induced HC of grade 2–4 in patients with cervical cancer.

## Research highlights

- First report of treatment for radiation-induced HC using intravesical instillation of KFL with thrombin and EGF.
- Safe therapeutic regimen for radiation-induced HC of grade 2-4 in patients with cervical cancer.
- Simple treatment methodology for radiation-induced HC with no adverse effects reported.

## References

[1] Irvin WP, Rice LW, Berkowitz RS. Advances in the management of endometrial adenocarcinoma. A review. J Reprod Med. 2002;47:173–189. 11933681

[2] Delaney G, Jacob S, Barton M. Estimation of an optimal radiotherapy utilization rate for gynecologic carcinoma: part I–malignancies of the cervix, ovary, vagina and vulva. Cancer. 2004;101:671–681. 15305396 10.1002/cncr.20444

[3] Smit SG, Heyns CF. Management of radiation cystitis. Nat Rev Urol. 2010;7:206–214. 20212517 10.1038/nrurol.2010.23

[4] Zwaans BM, Chancellor MB, Lamb LE. Modeling and treatment of radiation cystitis. Urology. 2016;88:14–21. 26571081 10.1016/j.urology.2015.11.001

[5] Kanai A, Epperly M, Pearce L, et al. Differing roles of mitochondrial nitric oxide synthase in cardiomyocytes and urothelial cells. Am J Physiol Heart Circ Physiol. 2004;286:H13–21. 14684357 10.1152/ajpheart.00737.2003

[6] Al Awamlh BAH, Lee DJ, Nguyen DP, et al. Assessment of the quality-of-life and functional outcomes in patients undergoing cystectomy and urinary diversion for the management of radiation-induced refractory benign disease. Urology. 2015;85:394–400. 25623700 10.1016/j.urology.2014.08.047

[7] Zwaans BMM, Lamb LE, Bartolone S, et al. Cancer survivorship issues with radiation and hemorrhagic systitis in gynecological malignancies. Int Urol Nephrol. 2018;50:1745–1751. 30132277 10.1007/s11255-018-1970-2 PMC6487476

[8] Manikandan R, Kumar S, Dorairajan LN. Hemorrhagic cystitis: a challenge to the urologist. Indian J Urol. 2010;26:159–166. 20877590 10.4103/0970-1591.65380 PMC2938536

[9] Sun XY. Shen Nong Ben Cao Jing. Shanghai: Commercial Press; 1955. p. 90.

[10] Luo TS, Gao MT, Ma FF, et al. Research advances in pharmacological action and clinical application of Periplaneta americana. J Anhui Agric Sci. 2012;40:5933–5935.

[11] Zhang HC, Geng FN, Shen YM, et al. Research progress of Kangfuxin Ye in pharmacological action and clinical application. Chin J Ethnomed Ethnopharm. 2016;26:57–60.

[12] Wen QC. Clinical analysis of Kangfuxin Liquid in prevention and treatment of acute radiation injury of oral mucosa reaction. J Clin Med Lit. 2014;1:467.

[13] Guo YH. Preventive effect of Kangfuxin liquid on perineal skin and mucosa injury in patients with cervical cancer after radiotherapy. Mod Med J China. 2017;19:70–71.

[14] Wu FF, Du X. Treatment of hemorrhagic cystitis after hematopoietic stem cell transplantation by intravesical instillation of Kangfuxin liquid: a randomized controlled study. Lab Med Clin. 2017;14:2766–2768.

[15] Sommariva ML, Sandri SD, Ceriani V. Efficacy of sodium hyaluronate in the management of chemical and radiation cystitis. Minerva Urol Nefrol. 2010;62:145–150. 20562794

[16] Zhu JJ, Shun Y, Guo X, et al. Bioactivity-guided screening of wound-healing active constituents from American cockroach (Periplaneta americana). Molecules. 2018;23:1. 10.3390/molecules23010101 PMC6017267 29361715

[17] Perez CA, Breaux S, Bedwinek JM, et al. Radiation therapy alone in the treatment of carcinoma of the uterine cervix. Cancer. 1984;54:235–246. 6722748 10.1002/1097-0142(19840715)54:2<235::aid-cncr2820540210>3.0.co;2-h

[18] Michael JZ, Heather C, Margie H, et al. Long-term outcome of high dose intensity modulated radiation therapy for patients with clinically localised prostate cancer. J Urol. 2006;176:1415–1419. 16952647 10.1016/j.juro.2006.06.002

[19] Noordzij JW, Dabhoiwala NF. Hemorrhagic radiation cystitis. Int Urogynecol J. 1993;4:160–167.

[20] Shao Y, Lu GL, Shen ZJ. Comparison of intravesical hyaluronic acid instillation and hyperbaric oxygen in the treatment of radiation-induced hemorrhagic cystitis. BJU Int. 2012;109:691–694. 21895939 10.1111/j.1464-410X.2011.10550.x

